# Consolidatory ablative stereotactic body radiation therapy after induction chemotherapy for unresectable pancreatic cancer: A single center experience

**DOI:** 10.3389/fonc.2022.974454

**Published:** 2022-11-18

**Authors:** Hye In Lee, Hyun-Cheol Kang, Eui Kyu Chie

**Affiliations:** ^1^ Department of Radiation Oncology, Seoul National University College of Medicine, Seoul, South Korea; ^2^ Department of Radiation Oncology, Seoul National University Hospital, Seoul, South Korea; ^3^ Institute of Radiation Medicine, Medical Research Center, Seoul National University, Seoul, South Korea

**Keywords:** pancreatic cancer, stereotactic body radiotherapy, ablative dose, MR-guided adaptive radiotherapy, simultaneous integrated protection, oligometastatic disease

## Abstract

**Background and purpose:**

Consolidatory radiotherapy in form of stereotactic body radiation therapy (SBRT) with an ablative dose following induction chemotherapy is emerging as a promising treatment scheme for unresectable pancreatic cancer. Outcomes of given treatment at a single center for contiguous patients with unresectable pancreatic cancer were evaluated to build the optimal treatment strategy.

**Materials and methods:**

In this retrospective study, a total of 50 patients with unresectable pancreatic cancer who underwent induction chemotherapy and ablative dose SBRT were included. SBRT dose was 40–50 Gy in five fractions. Two strategies were adopted to adhere to the organs at risk (OAR) dose constraints: simultaneous integrated protection (SIP) technique and magnetic resonance (MR)-guided adaptive technique. Overall survival (OS) and local progression-free survival (LPFS) were calculated from the start date of SBRT.

**Results:**

The median follow-up period for survivors was 21.1 months (range, 6.2–61.0 months). Eleven (22.0%) patients underwent resection after SBRT, which were all R0 resection. In patients with non-metastatic disease, the median OS was 26.5 months (range, 4.1–61.0 months), and the 1- and 3-year LPFS were 90.0% (95% confidence interval [CI], 72.0–96.7%) and 57.4% (95% CI, 31.7–76.4%), respectively. Patients with oligometastatic disease had inferior survival outcomes, but there was no survival difference among responders to induction chemotherapy. In the multivariable analysis, tumor size ≤4 cm, non-metastatic status, and good response to induction chemotherapy were associated with improved LPFS. In dosimetric analysis, GTV Dmin ≥50.5 Gy was the strongest prognosticator against local progression. Grade ≥3 adverse events occurred in two (4.0%) patients with non-adaptive RT, but none in patients with MR-guided adaptive RT.

**Conclusion:**

Ablative dose SBRT following induction chemotherapy is an effective strategy for selected patients with unresectable pancreatic cancer. The SIP technique and MR-guided adaptive RT were attributed to minimizing the risk of adverse events. Further studies are needed to identify the best candidates for consolidatory SBRT in unresectable pancreatic cancer.

## Introduction

Pancreatic cancer is one of the leading causes of cancer-related mortality worldwide. Most patients with pancreatic cancer present with locally advanced or metastatic disease and are not amenable to curative surgery (1). Chemotherapy is considered the standard of care for these patients, but the prognosis remains dismal, with a 5-year life expectancy of less than 10% (2). Recently, new multi-agent chemotherapy regimens have changed this paradigm (3–5). The combination regimen of 5-fluorouracil, leucovorin, irinotecan, and oxaliplatin (FOLFIRINOX) and gemcitabine with nanoparticle albumin-bound-paclitaxel (Gem/nab-paclitaxel) have become the first-line treatment options for unresectable pancreatic cancer. These regimens have been demonstrated to almost double the survival of unresectable pancreatic cancer compared with the previous monochemotherapy regimens. However, the limited gain in terms of local control may possibly expand the role of RT for local control.

Stereotactic body radiation therapy (SBRT) has emerged as an attractive approach in multimodality treatment for pancreatic cancer (6–8). SBRT offers the ability to deliver a large biologically effective dose (BED) in a highly conformal manner. Furthermore, SBRT interferes less with systemic therapy as it requires only 1–2 weeks for delivery. The optimal SBRT scheme has yet to be determined, but the administration of a higher BED is essential to achieve durable tumor control and has a significant impact on survival (9). However, SBRT for pancreatic cancer is challenging due to the proximity of radiosensitive organs-at-risk (OAR), such as the duodenum, stomach, and bowel. Although prospective data regarding pancreatic SBRT is accumulating, the optimal treatment strategy remains controversial (10).

In this study, simultaneous integrated protection (SIP) technique and magnetic resonance (MR)-guided adaptive technique are implemented as components of SBRT to deliver ablative dose SBRT while minimizing the treatment-related adverse event risk. The resultant efficacy and safety of the applied treatment is evaluated. In addition, we investigated optimal SBRT strategies, including patient selection and dosimetric parameters.

## Materials and methods

### Study cohort

**Figure 1:**
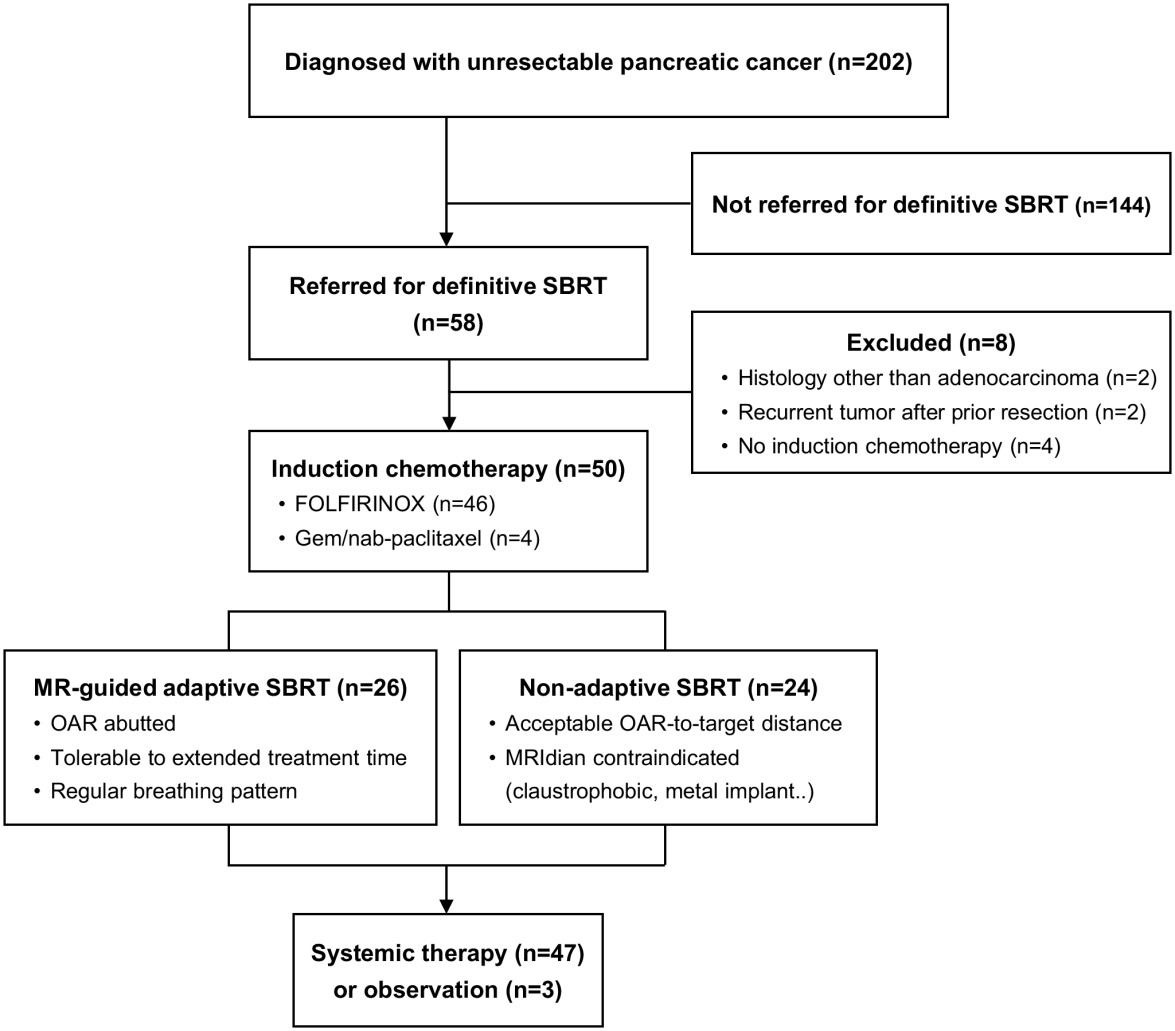
Consort diagram. MR, magnetic resonance; SBRT, stereotactic body radiotherapy; OAR, organs at risk.

We retrospectively identified patients with locally advanced pancreatic adenocarcinoma, including oligometastatic disease, who received induction chemotherapy followed by ablative dose SBRT between January 2017 and September 2021. Locally advanced stage was defined as a tumor with greater than 180-degree involvement of the superior mesenteric artery or celiac axis or unreconstructable involvement of the superior mesenteric vein or portal vein. Oligometastatic disease was defined as less than four metastases to a single organ. Patients were excluded if they had a prior definitive treatment history, pancreatic tumor histology other than adenocarcinoma, or double-primary malignancies. A total of 50 patients with unresectable pancreatic cancer constituted the analyzed cohort (**Figure 1**). This study was approved by the Institutional Review Board (IRB no. H-2105-070-1218).

### Treatment

All patients received four or more cycles of induction chemotherapy before SBRT. Induction chemotherapy consisted of FOLFIRINOX (92.0%) and Gem/nab-paclitaxel (8.0%) regimens. A 3–4 week break from induction chemotherapy was required before SBRT delivery.

SBRT was performed in two ways; MR-guided adaptive SBRT was delivered using the MRIdian (ViewRay Inc., Oakwood Village, OH) and non-adaptive linear accelerator (linac)-based SBRT was delivered using the TrueBeam-STX (Varian Medical Systems, Palo Alto, CA). Patients underwent both MR and computed tomography (CT) simulations on the same day. Patients were immobilized in the supine position with arms over head. A pneumatic abdominal compressor was used to reduce breathing-induced internal tumor movement. Patients who were unable to breathe regularly, could not tolerate extended treatment times, or had any contraindications to MR (e.g., claustrophobic, metal implant) were assigned to non-adaptive SBRT. Otherwise, both adaptive and non-adaptive SBRT plans were constructed, and the best plan for an individual patient was chosen based on both OAR dose and target coverage.

**Figure 2:**
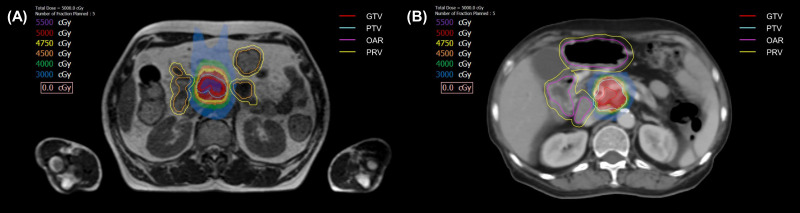
The target volumes and isodose lines for patients prescribed 50 Gy in 5 fractions with magnetic resonance-guided adaptive radiotherapy **(A)**, and linac-based nonadaptive radiotherapy **(B)**. GTV, gross tumor volume; PTV, planning target volume; OAR, organ at risk; PRV, planning OAR volume.

The principle of tumor delineation was same for both set-up (**Figure 2**). Gross tumor volume (GTV) was defined as pancreatic tumor based on 4-dimensional–CT and MR images. Clinical target volume (CTV) encompassed the GTV and vascular involvement, including the entire tumor-vessel interface. Planning target volume (PTV) included the CTV plus a 6 mm margin. Planning OAR volume (PRV) was defined as OAR plus a 4 mm margin. If the patient had an overlapping area between PTV and PRV, PTV was divided into two; PTV_sip_ was the overlap area between PTV and PRV, and PTV_tumor_ was the remaining, non-overlapping PTV area with PRV. Using the SIP technique, we prescribed 33 Gy to the PTV_sip_, while simultaneously delivering a 50 Gy to the PTV_tumor_. Exceptionally, GTV was prescribed to 50 Gy even if there was an overlapping area with PRV as long as OAR constraints, which was <33 Gy to 1cc, were met. Prescribed doses were lowered down to 40 Gy depending on the situation, including target/organ movement and target to organ distance of the individual patient. SBRT was delivered in five fractions with the goal of 95% PTV coverage with 100% of the prescription dose, prioritizing hard OAR constraints. OAR dose constraints were as follows: for the duodenum, bowel, and stomach, Dmax <35 Gy and D1cc <33 Gy; for the spinal cord, Dmax <22 Gy; for kidneys, Dmean <10 Gy; and for the liver, 700 cm3 <21 Gy. If the OAR-to-target distance was sufficient and the PTV coverage and OAR constraints were simultaneously met, the patient was often assigned to non-adaptive linac-based SBRT, due to the convenience of a shorter treatment delivery time. In contrast, if PTV coverage could not be met due to a violation of OAR constraints, the patient was usually assigned to MR-guided adaptive SBRT.

For adaptive SBRT delivery, MR imaging was performed before each fraction, and new OARs were re-contoured, reflecting inter-fractional changes. Then, the new re-optimized plan was generated. If the new plan violated OAR constraints, plan normalization was altered till OAR constraints were fulfilled. After the daily adaptive plan was determined, each fraction was delivered under MR-guided real-time gating. In non-adaptive SBRT, image-guided RT using cone beam CT was performed for all fractions. To reduce inter- and intra-fractional variability, a 6-hour fasting period before simulation and each fraction was mandated.

### Follow-up

After completing SBRT, physical examination, laboratory tests, and imaging were performed every 2–6 months or when clinically indicated. Most patients continued to receive additional systemic therapy. The multidisciplinary tumor board determined resectability after reviewing the imaging and performance status. Adverse events were graded using the Common Terminology Criteria for Adverse Events (version 5.0). Acute adverse events were defined as those occurring within three months after SBRT, whereas late adverse events were defined as those occurring beyond three months after SBRT.

### Statistical analysis

Response evaluation was performed using response evaluation criteria in solid tumors (RECIST). The response to induction chemotherapy was evaluated in two ways. The overall response was measured by comparing the disease status between the time of initial diagnosis and the time of SBRT. Whereas, the response at SBRT referral compared disease status between three months before SBRT and at the time of SBRT. For dosimetric analysis, all parameters were depicted from the cumulative daily delivered re-optimized plans for the adaptive treatments, whereas the original treatment plan was used for the non-adaptive treatments. GTV Dmean was defined as the mean dose absorbed by the GTV. GTV Dmin and GTV Dmax were defined as the minimum and maximum dose absorbed by 1 cc of GTV, respectively.

Local progression was defined as a 20% or more increase in tumor size on the CT scan compared to previous imaging following the RECIST criteria. Local progression-free survival (LPFS) was calculated from the start date of SBRT to the date of local progression or death. Overall survival (OS) was calculated from the start date of SBRT to the date of death. Surviving patients were censored at the date of the last follow-up. Survival outcomes were estimated using the Kaplan–Meier method. Cox proportional hazards modeling assessed whether survival outcomes varied according to risk factors. Continuous variables, including dose parameters, were divided into two subgroups at cutoff values identified by receiver operating characteristic (ROC) curves and then analyzed. All statistical tests were performed using STATA (version 15.1; StataCorp LP, College Station, TX, USA).

## Results

### Patient and treatment characteristics

Patients and treatment characteristics are summarized in **Table 1**. The median age was 62 years (range, 39–78 years), and the median tumor size was 3.0 cm (range, 1.6–5.5 cm). A total of 16 (32.0%) patients had nodal involvement and 17 (34.0%) patients had oligometastatic lesion. After induction chemotherapy for a median of 13 cycles (range, 4–29 cycles), the overall response was a partial response (PR) in 28 (56.0%), stable disease (SD) in 18 (36.0%), and progressive disease (PD) in 4 (8.0%) patients. The response at SBRT referral included fewer PR’s and more SD’s and PD’s (PR: 44.0%, SD: 42.0%, and PD: 14.0%, respectively). The median prescribed dose was 50.0 Gy (range, 40–50 Gy), and the SIP protocol was applied in 29 (58%) patients. Of the 26 patients (52.0%) who received MR-guided adaptive SBRT, the delivered dose was reduced in 16 patients (32.0%) to abide by the OAR constraint. A total of 47 (94.0%) patients received post-SBRT chemotherapy for a median number of 8 cycles (range, 1–41 cycles).

**Table 1:** Patients and treatment characteristics (N=50).

Variable	No. (%)	
Age at diagnosis (years), median	62	(39–78)
Gender		
Male	22	(44.0%)
Female	28	(56.0%)
ECOG performance status		
ECOG 0-1	32	(64.0%)
ECOG 2-3	18	(36.0%)
Location of tumor		
Head	21	(42.0%)
Body/tail	29	(58.0%)
Tumor size at diagnosis (cm), median	3.0	(1.6–5.5)
Tumor size at SBRT (cm), median	2.4	(1.0–4.5)
Clinical T-stage		
T2	6	(12.0%)
T3	2	(4.0%)
T4	42	(84.0%)
Clinical N-stage		
N0	34	(68.0%)
N1	16	(32.0%)
Clinical M-stage		
M0	33	(66.0%)
M1	17	(34.0%)
Induction CRx regimen		
FOLFIRINOX	46	(92.0%)
Gemcitabine and nab-paclitaxel	4	(8.0%)
Induction CRx duration (mo), median	7.2	(1.5–21.9)
No. of induction CRx cycles, median	13	(4–29)
CA 19-9, baseline (U/mL), median	370	(2 to >12000)
CA 19-9, post-induction CRx/pre-SBRT (U/mL), median	36	(1 to >12000)
CA 19-9 post-SBRT (U/mL), median	19	(1 to >12000)
SBRT technique		
MR-guided adaptive SBRT	26	(52.0%)
Linac based non-adaptive SBRT	24	(48.0%)
Prescribed dose (Gy), median	50.0	(40–50)
GTV Dmin (Gy)^1^, median	50.3	(40.8–58.3)
GTV Dmax (Gy)^2^, median	51.8	(43.8–68.4)
GTV Dmean (Gy)^3^, median	51.0	(42.4–62.1)
GTV volume (cm^3^), median	13.6	(2.8–54.8)
PTV volume (cm^3^), median	33.6	(9.0–107.0)
PTV_sip_ volume (cm^3^), median	1.2	(0–6.4)

No, patients’ number; SBRT, stereotactic body radiotherapy; ECOG, Eastern Cooperative Oncology Group; CRx, chemotherapy; mo, months; MR, magnetic resonance; Linac, linear accelerator; GTV, gross tumor volume; PTV, planning target volume; SIP, simultaneous integrated protection.

^1^GTV Dmin, the minimum dose absorbed by 1cc of the GTV. ^2^GTV Dmax, the maximum dose absorbed by 1cc of the GTV. ^3^GTV Dmean, mean dose absorbed by the GTV.

A dramatic decline in carbohydrate antigen (CA) 19-9 was observed after induction chemotherapy and SBRT. The median % change of CA 19-9 was -82.7% from baseline to post-induction chemotherapy/pre-SBRT and -38.8% from post-induction chemotherapy/pre-SBRT to post-SBRT.

### Treatment outcomes

**Figure 3:**
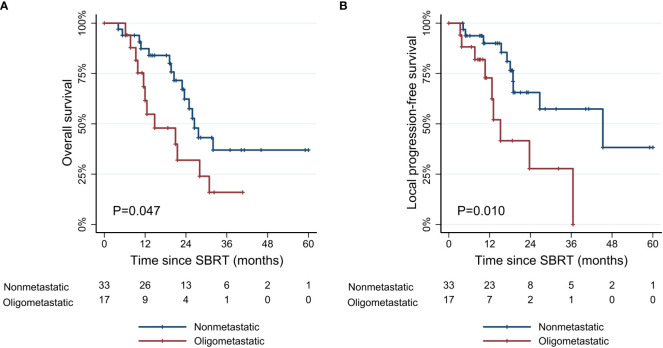
Kaplan-Meier curves of **(A)** overall survival, and **(B)** local progression-free survival according to metastatic status. SBRT, stereotactic body radiotherapy.

The median follow-up period was 18.8 months (range, 4.1–61.0 months) for all patients and 21.1 months (range, 6.2–61.0 months) for survivors from the start date of SBRT. In patients with non-metastatic disease, the median OS was 26.5 months (range, 4.1–61.0 months), and the 1- and 3-year OS rates were 87.3% (95% confidence interval [CI], 69.6–95.1%) and 37.0% (95% CI, 17.4–56.8%), respectively. Patients with oligometastatic disease had inferior survival outcomes with a median OS of 12.5 months (range, 6.2–40.7 months), and the 1- and 3-year OS rates of 61.6% (95% CI, 33.5–80.7%) and 16.0% (95% CI, 2.7–39.5%), respectively (**Figure 3**). However, this difference diminished in patients responding to induction chemotherapy (oligometastatic/PR: 1-year OS 85.7% [95% CI, 33.4–97.9%] and 3-year OS 35.7% [95% CI, 5.2–69.9%]; oligometastatic/SD-PD: 1-year OS 40.5% [95% CI, 10.0–70.1%] and 3-year OS 0%). The 1- and 3-year LPFS rates of the non-metastatic group were 90.0% (95% CI, 72.0–96.7%) and 57.4% (95% CI, 31.7–76.4%), respectively. In oligometastatic/PR group, the 1- and 3-year LPFS were 100.0% and 44.4% (95% CI, 6.6–78.5%), respectively. Meanwhile, the corresponding rates of oligometastatic/SD-PD group were 45.7% (95% CI, 8.2–78.3%) and 0%, respectively.

After SBRT, eleven (22.0%) patients underwent resection, and all had R0 resection, with three (6.0%) achieving a pathologic complete response (pCR). There were no grade ≥3 postoperative adverse events. Patients with resected tumors showed significantly improved survival outcomes compared with patients without resection (1-year OS: 90.9% [95% CI, 50.8–98.7%] vs. 75.1% [95% CI, 57.5–86.2%]; 3-year OS: 64.9% [95% CI, 24.9–87.4%] vs. 19.0% [95% CI, 6.7–36.0%]; p=0.014). At the last follow-up, 16 (32.0%) patients were in a progression-free state with normalized CA 19-9 and without imaging evidence of progression.

### Prognostic factors

The univariate and multivariate Cox proportional hazards models for OS and LPFS are presented in **Table 2**. In the multivariate analysis, M-stage was a strong prognostic factor for both OS and LPFS (Hazard ratio [HR] 2.74, p=0.018 in OS; HR 5.32, p=0.003 in LPFS). A large initial tumor size (≥4 cm) was associated with inferior LPFS (HR 4.26, p=0.003). Response to the induction chemotherapy in overall (HR 3.79, p=0.044) and at SBRT referral (HR 4.27, p=0.001) were both significantly related to OS, but only response at SBRT referral was a significant factor for LPFS (HR 3.72, p=0.022). Among dose parameters, higher GTV Dmin (≥50.5 Gy) was significantly associated with improved LPFS (HR 3.06, p=0.045) and OS (HR 2.58, p=0.031). However, the prescribed dose (≥50 Gy), GTV Dmax (≥52 Gy), and GTV Dmean (≥51 Gy) failed to demonstrate statistical significance. Greater CA 19-9 decline after SBRT (≥50%) had a strong relationship with improved LPFS (HR 3.85, p=0.015). On the other hand, the volume of PTV and PTV_sip_ did not affect survival outcomes. SBRT technique (adaptive vs. non-adaptive) also had no significant association with both OS and LPFS.

**Table 2:** Univariate and multivariate analysis affecting overall survival and local progression-free survival.

	Overall survival						Local progression-free survival					
Variables	Univariate			Multivariate			Univariate			Multivariate		
	HR	95% CI	p-value	HR	95% CI	p-value	HR	95% CI	p-value	HR	95% CI	p-value
**Baseline characteristic**												
Age at diagnosis (>60 vs. ≤60)	1.47	0.68-3.16	0.324				0.93	0.39-2.37	0.925			
Sex (male vs. female)	0.78	0.36-1.68	0.525				0.46	0.17-1.22	0.119			
Performance status (ECOG 2/3 vs. ECOG 0/1)	1.40	0.65-2.99	0.390				1.00	0.39-2.55	0.996			
Tumor location (head vs. body/tail)	1.46	0.67-3.17	0.340				1.03	0.40-2.62	0.956			
Tumor size (>4 cm vs. ≤4 cm)	1.29	0.60-2.79	0.518				3.20	1.28-7.98	**0.013**	4.26	1.61-11.24	**0.003**
T-stage (T4 vs. T1-T3)	1.12	0.39-3.25	0.833				5.59	0.71-44.2	0.103			
N-stage (N1 vs. N0)	1.05	0.47-2.33	0.914				0.84	0.32-2.23	0.735			
M-stage (M1 vs. M0)	2.13	0.99-4.57	**0.052**	2.74	1.19-6.29	**0.018**	3.23	1.27-8.25	**0.014**	5.32	1.76-16.08	**0.003**
CA 19-9 (>37.0 vs. ≤37 U/mL)	0.58	0.22-1.54	0.274				3.61	0.48-27.20	0.214			
**Induction CRx**												
Induction CRx regimen (FOLFIRINOX vs. Gem/nab-paclitaxel)	0.99	0.23-4.18	0.985				1.18	0.36-3.87	0.788			
Induction CRx duration (≥3 months vs. <3 months)	4.36	1.22-15.57	**0.023**	3.79	1.03-13.93	**0.044**	2.12	0.64-7.03	0.220			
Response to induction CRx, overall (SD/PD vs. PR)	3.41	1.55-7.50	**0.002**	4.27	1.82-10.02	**0.001**	1.50	0.57-3.94	0.415			
Response to induction CRx at SBRT referral (SD/PD vs. PR)	2.62	1.16-5.88	**0.020**	3.22	1.33-7.79	**0.010**	2.27	0.86-6.01	**0.093**	3.72	1.21-11.43	**0.022**
CA19-9% change after induction CRx (<50% vs. ≥50%)	1.48	0.62-3.54	0.378				1.45	0.68-3.10	0.331			
**SBRT**												
SBRT technique (adaptive vs. non-adaptive)	1.20	0.54-2.67	0.659				1.87	0.73-4.78	0.190			
Prescribed dose (<50 Gy vs. ≥50 Gy)	2.65	1.21-5.83	**0.015**	2.17	0.97-4.84	0.060	2.44	0.95-6.27	**0.065**	1.96	0.75-5.10	0.169
GTV Dmin^1^ (<50.5 Gy vs. ≥50.5 Gy)	2.30	1.01-5.25	**0.049**	2.58	1.09-6.09	**0.031**	4.18	1.48-11.82	**0.007**	3.06	1.03-9.14	**0.045**
GTV Dmax^2^ (<52 Gy vs. ≥52 Gy)	1.47	0.67-3.23	0.335				2.03	0.76-5.46	0.158			
GTV Dmean^3^ (<51 Gy vs. ≥51 Gy)	1.22	0.57-2.65	0.608				1.92	0.75-4.93	0.173			
PTV volume (<23 cm^3^ vs. ≥23 cm^3^)	0.72	0.27-1.92	0.516				0.47	0.14-1.62	0.262			
PTV_sip_ volume (<1.6 cm^3^ vs. ≥1.6 cm^3^)	0.81	0.30-2.13	0.659				0.43	0.10-1.85	0.256			
CA19-9% change after SBRT (<50% vs. ≥50%)	2.90	1.25-6.71	**0.013**	2.25	0.80-6.34	0.123	3.86	1.32-11.27	**0.014**	3.85	1.31-11.35	**0.015**
**Post-SBRT CRx**												
Post-SBRT CRx regimen (FOLFIRINOX vs. others)	0.55	0.23-1.33	0.182				1.34	0.44-4.05	0.610			

ECOG, Eastern Cooperative Oncology Group; CRx, chemotherapy; Gem/abraxane, gemcitabine + nab-paclitaxel; SD, stable disease; PD, progressive disease; PR, partial response; SBRT, stereotactic body radiotherapy; GTV, gross tumor volume; PTV, planning target volume; SIP, simultaneous integrated protection; HR, hazard ratio; CI, Confidence interval.

^1^GTV Dmin, the minimum dose absorbed by 1cc of the GTV. ^2^GTV Dmax, the maximum dose absorbed by 1cc of the GTV. ^3^GTV Dmean, mean dose absorbed by the GTV. p-values below 0.05 are shown in bold.

### Dosimetric analysis

**Figure 4:**
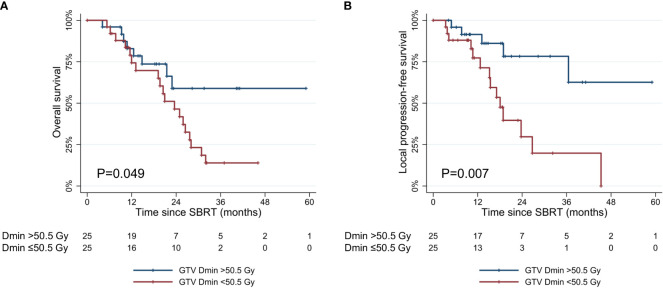
Kaplan-Meier curves of **(A)** overall survival, and **(B)** local progression-free survival according to GTV Dmin (≥50.5 Gy vs. <50.5 Gy). SBRT, stereotactic body radiotherapy; GTV, gross tumor volume. GTV Dmin = the minimum dose absorbed by 1cc of the GTV.

The values of dosimetric parameters in patients with or without local progression within three years following SBRT are shown in **Table 3**. The mean values were all higher in patients without local progression compared to those with local progression. Among dosimetric parameters, the difference between the two groups was significant for the prescribed dose (p=0.006), GTV Dmin (p<0.001), and GTV Dmean (p=0.008). According to the ROC analysis, GTV Dmin showed the highest AUC (0.789) as a predictor of local progression (**Supplementary Figure 1**). Patients with GTV Dmin ≥50.5 Gy showed superior OS and LPFS compared to those with GTV Dmin < 50.5 Gy (1-year OS: 82.9% [95% CI, 60.6–93.2%] vs. 74.3% [95% CI, 51.4–87.6%], p=0.049; 1-year LPFS: 91.5% [95% CI, 70.0–97.8%] vs. 77.3% [95% CI, 53.2–54.2%], p=0.007) (**Figure 4**).

**Table 3:** Comparison of dosimetric parameters between patient groups with or without local progression within 3 years following SBRT.

	No local progression (N=33)	Local progression (N=17)	P-value	AUC
Prescribed dose (Gy)	48.7 ± 2.2	46.3 ± 3.9	0.006	0.664
GTV Dmin (Gy)	51.3 ± 0.5	47.8 ± 4.2	<0.001	0.789
GTV Dmax (Gy)	55.3 ± 0.9	52.1 ± 1.5	0.063	0.717
GTV Dmean (Gy)	53.4 ± 3.7	50.0 ± 1.1	0.008	0.735

SBRT, stereotactic body radiotherapy; GTV, gross tumor volume; AUC, area under curve.

GTV Dmin, the minimum dose absorbed by 1cc of the GTV; GTV Dmax, the maximum dose absorbed by 1cc of the GTV; GTV Dmean, mean dose absorbed by the GTV.

*Data are expressed as mean ± standard deviation.

### Adverse events

The acute and late adverse events are presented in **Table 4**. Acute grade 2 adverse events occurred in 23 (46.0%) patients. There were no grade 3 or higher acute adverse events. The most common acute adverse events were abdominal pain (32.0%), nausea/vomiting (28.0%), and poor oral intake (16.0%). Regarding the late adverse events, grade 2 or higher events occurred in 15 (30.0%) patients. A total of two (6.0%) patients experienced grade 3 gastro-intestinal bleeding, both requiring endoscopic intervention, but were successfully managed. There were no grade 4 or higher late adverse events. Patients treated with MR-guided adaptive SBRT showed a trend toward lower rates of late adverse events compared with those treated with non-adaptive SBRT (grade 2: 19.2% vs. 33.3%; grade 3: 0% vs. 8.3%; p=0.084).

**Table 4:** Acute and late adverse events after SBRT.

	Grade 2		Grade 3	
Acute				
Abdominal pain	16	(32.0%)	0	(0.0%)
Nausea/Vomiting	14	(28.0%)	0	(0.0%)
Poor oral intake	8	(16.0%)	0	(0.0%)
Diarrhea	3	(6.0%)	0	(0.0%)
Total	23	(46.0%)	0	(0.0%)
Chronic				
Abdominal pain	6	(12.0%)	1	(2.0%)
Bleeding	6	(12.0%)	2	(4.0%)
Ulcer	4	(8.0%)	2	(4.0%)
Gastritis	3	(6.0%)	0	(0.0%)
Fistula	1	(2.0%)	0	(0.0%)
Total	13	(26.0%)	2	(4.0%)

*No grade 4 or 5 adverse events.

## Discussion

Pancreatic cancer is perceived as a systemic disease with the eventual emergence of widespread metastases. Despite this, up to 30% of patients with pancreatic cancer are reported to be without metastatic disease at the time of death (11). This percentage is likely to further increase as systemic therapy is continuously advancing in both multiagent cytotoxic approaches and combined precision medicine-based strategies (4, 12). In an era of more effective systemic therapy, maximizing local treatment has become more important, which may eventually lead to improved treatment outcomes. However, the use of conventionally fractionated RT failed to convey survival benefits in addition to the standard of care chemotherapy in a prospective trial, despite improvement in local control (13).

In this context, SBRT has emerged as an attractive alternative showing the potential to improve local control, with an ability to deliver a higher dose in a conformal manner and requiring shorter overall treatment time, simultaneously. A recent meta-analysis reported that SBRT significantly improved 2-year OS compared to conventionally fractionated RT (26.9% vs. 13.7%, p = 0.004) (6). Petrelli et al. performed a systemic review of 19 trials of SBRT and reported the pooled 1-year OS rate of 51.6% (95% CI 41.4-61.7%) and 1-year local control rate of 72.3% (95% CI 58.5-79%) (14). Many of these data suggest that a higher dose is needed to achieve adequate tumor control (15, 16). Toesca et al. reviewed the treatment outcomes of 149 patients who received multi-fraction SBRT for unresectable pancreatic cancer (15). They reported that patients treated with SBRT dose ≥40 Gy had superior OS and PFS compared to those who received SBRT dose <40 Gy (median OS: 23 vs. 14 months, p=0.0007; median PFS: 13 vs. 10 months, p = 0.007). In this study, we reported the 1-year OS and LPFS rates of 87.3% and 90.0%, respectively, which is in line with SBRT series with an ablative-dose (BED_10_ ≥100 Gy). However, SBRT delivering an ablative dose is still limited due to the proximity of critical neighboring radiosensitive OARs. Furthermore, the tumor and surrounding structures are highly mobile and sometimes difficult to identify using cone beam CT-based imaging. Thus, in this study, we adopted two approaches to safely deliver ablative doses.

The SIP technique allows the simultaneous delivery of ablative doses to the tumor volume, whereas the overlapping volume with critical OARs is covered by a lowered, safer dose (17). Several studies have reported the results of SIP protocol in different RT schemes (18–20). Simoni et al. performed SBRT using the SIP technique by administering 50 Gy to the tumor-vessel interface (TVI), 30 Gy to the pancreatic tumor, and 25 Gy to the SIP volume (18). They found no acute or late grade ≥3 adverse events, but a predominant incidence of in-field failures occurred. After that, the authors investigated dose escalation protocols up to 60, 40, and 33 Gy to TVI, pancreatic tumor, and SIP volume, respectively, and demonstrated the feasibility with adequate PTV coverage and acceptable OAR exposure (21). In this cohort, we prescribed 50 Gy to the TVI and tumor and 33 Gy to the SIP volume. We found favorable toxicity profiles without compromising survival outcomes. The volume of PTV and PTV_sip_ did not affect survival outcomes in multivariable analysis, although the small cohort size may have obscured the impact.

On the other hand, MR-guided adaptive RT is another powerful tool ensuring accurate treatment delivery with several advantages (22). First, MR guidance offers improved soft tissue contrast, resulting in the ability to distinguish the boundaries of pancreatic tumors. Second, a new re-optimized plan can be generated and optimized per fraction, reflecting daily anatomical changes. Hassanzadeh et al. found that duodenal dose constraints would have been violated in 67.7% of fractions for pancreatic cancer patients without per fraction optimization (23). Third, a real-time gating system enables intra-treatment monitoring of OARs. Several retrospective studies of MR-guided adaptive RT have reported promising outcomes while minimizing toxicities (24, 25). In the current study, grade ≥ 3 adverse events occurred in two (4.0%) patients with non-adaptive RT, and none in patients with MR-guided adaptive RT. It is notable that patients with close OAR-to-target distance were mostly assigned to MR-guided adaptive SBRT (**Figure 1**). Therefore, MR-guided adaptive RT may be more suitable for patients with a high risk of adverse events. Given the low conversion rate to surgery, safety concerns should be a top priority for SBRT treatment. The adoption of SIP technique and MR-guided adaptive RT can contribute to minimizing the risk of adverse events while delivering ablative doses to the tumor.

Currently, the optimal SBRT strategies have yet to be determined for pancreatic cancer. Published studies have used various combinations of dose and fraction schemes. Nonetheless, in order to perform SBRT effectively, particularly in daily adaptive SBRT, it is essential to identify significant dose parameters. Current dosimetric analysis nominated GTV Dmin as the most relevant parameter against local progression, with the cutoff value of 50.5 Gy, as determined by ROC analysis. Thus, patients with smaller PTV and PTV_sip_ volumes may be better candidates for SBRT that meet the dosimetric requirements and minimize adverse events. In addition, our results found that patients who had a tumor size ≤4 cm, non-metastatic status, and good response to induction chemotherapy showed a better prognosis after SBRT. Response to induction chemotherapy at three months prior to SBRT was more predictive of treatment outcomes compared to at the time of diagnosis. This may reflect that prompt delivery of consolidatory treatment would be a more effective strategy for patients with an initial response to induction chemotherapy but a stationary response afterward, compared with continued chemotherapy till progression.

Furthermore, it is notable that patients who had oligometastatic disease but achieved response to induction chemotherapy showed similar survival rates compared to non-metastatic patients in this study. Only few studies have examined the role of SBRT in metastatic pancreatic cancer. Lischalk et al. evaluated 20 patients with metastatic pancreatic cancer who received chemotherapy and SBRT (26). They reported 1-year OS and local control rates of 43% and 53%, respectively, without grade ≥3 toxicities. They also found that smaller PTV was associated with improved OS (p=0.001) and local control rates (p=0.02). Rosati et al. recommended a minimum of 6 months of chemotherapy and an observation period (4–8 weeks) before SBRT to better understand the natural history of disease (27). In this study, we found that a longer duration of induction chemotherapy (≥3 months) and good response to chemotherapy were associated with improved survival outcomes. Although based on the observation from very selected small population, SBRT to the primary site may have a role for oligometastatic patients who underwent chemotherapy over a period of time and achieve a durable response to induction chemotherapy. On the contrary, we do not advocate SBRT for patients with oligometastatic disease who did not respond to induction chemotherapy, as patients with these unfavorable factors eventually experienced disease progression within six months from SBRT. This principle is in line with selectively offering ablative local treatment in oligo-persistent disease.

There are several limitations to this study. First, the number of patients is far insufficient to draw concrete conclusion, especially in the oligometastatic disease subgroup. Additionally, as this was a retrospective study, the events could have been underestimated due to incomplete medical records. Second, the dosimetric analysis might be biased because lower doses were prescribed in patients with poor performance status. Finally, MR-guided adaptive SBRT was performed using the Cobalt-60 system in this study. Better OAR sparing may be achieved with MR-linac system with a steeper dose gradient. Nonetheless, the entire cohort was treated with the uniform treatment protocol, including dose prescription, target delineation, and decision policy for radiotherapy technique. Furthermore, they all received FOLFIRINOX or Gem/nab-paclitaxel, which are currently considered the standard of care as the first-line treatments. Thus, our findings may have better applicability and generalizability to current clinical practice.

In conclusion, consolidatory ablative dose SBRT following induction chemotherapy could be a viable treatment option for selected patients with unresectable pancreatic cancer. Patients with a tumor size ≤4 cm and achieved a durable response to induction chemotherapy may be good candidates for SBRT treatment as consolidatory measure. Prompt ablative consolidatory treatment delivery may be more appropriate approach compared to sustained chemotherapy beyond initial response. For SBRT planning, GTV Dmin (≥50.5 Gy) was identified as the most relevant parameter against local progression. The SIP technique and MR-guided adaptive RT strategies enabled the delivery of an ablative dose to the tumor while minimizing toxicity of surrounding OARs. Further studies with larger cohort sizes, better yet prospective design would help to further validate the role of the optimal SBRT strategies in pancreatic cancer.

## Data availability statement

The raw data supporting the conclusions of this article will be made available by the authors, without undue reservation.

## Ethics statement

The studies involving human participants were reviewed and approved by The Institutional Review Board of Seoul National University Hospital (IRB no. H-2105-070-1218). Written informed consent for participation was not required for this study in accordance with the national legislation and the institutional requirements.

## Author contributions

EC contributed conception and design of the study. HL contributed to data collection, statistical analysis and wrote the first draft of the manuscript. EC and H-CK revised the manuscript. All authors contributed to the article and approved the submitted version.

## Conflict of interest

The authors declare that the research was conducted in the absence of any commercial or financial relationships that could be construed as a potential conflict of interest.

## Publisher’s note

All claims expressed in this article are solely those of the authors and do not necessarily represent those of their affiliated organizations, or those of the publisher, the editors and the reviewers. Any product that may be evaluated in this article, or claim that may be made by its manufacturer, is not guaranteed or endorsed by the publisher.

## Supplementary material

Supplementary Figure 1 Kaplan-Meier curves of **(A)** overall survival, and **(B)** local progression-free survival according to metastatic status and overall response to the induction chemotherapy. SBRT, stereotactic body radiotherapy.

Supplementary Figure 2 Comparison of receiver operating characteristic (ROC) curves of dosimetric parameters for predicting the 3-year incidence of local progression. GTV, gross tumor volume; AUC, area under curve.GTV Dmin, the minimum dose absorbed by 1cc of the GTV; GTV Dmax, the maximum dose absorbed by 1cc of the GTV; GTV Dmean, mean dose absorbed by the GTV.

## References

[1] McGuigan A Kelly P Turkington RC Jones C Coleman HG McCain RS. Pancreatic cancer: A review of clinical diagnosis, epidemiology, treatment and outcomes. World J Gastroenterol (2018) 24:4846–61. doi: 10.3748/wjg.v24.i43.4846 PMC6250924 30487695

[2] Siegel RL Miller KD Fuchs HE Jemal A. Cancer statistics, 2021. CA Cancer J Clin (2021) 71:7–33. doi: 10.3322/caac.21654 33433946

[3] Conroy T Desseigne F Ychou M Bouché O Guimbaud R Bécouarn Y. FOLFIRINOX versus gemcitabine for metastatic pancreatic cancer. N Engl J Med (2011) 364:1817–25. doi: 10.1056/nejmoa1011923 21561347

[4] Suker M Beumer BR Sadot E Marthey L Faris JE Mellon EA. FOLFIRINOX for locally advanced pancreatic cancer: a systematic review and patient-level meta-analysis. Lancet Oncol (2016) 17:801–10. doi: 10.1016/S1470-2045(16)00172-8 PMC5527756 27160474

[5] Von Hoff DD Ervin T Arena FP Chiorean EG Infante J Moore M. Increased survival in pancreatic cancer with nab-paclitaxel plus gemcitabine. N Engl J Med (2013) 369:1691–703. doi: 10.1056/nejmoa1304369 PMC4631139 24131140

[6] Tchelebi LT Lehrer EJ Trifiletti DM Sharma NK Gusani NJ Crane CH. Conventionally fractionated radiation therapy versus stereotactic body radiation therapy for locally advanced pancreatic cancer (CRiSP): An international systematic review and meta-analysis. Cancer (2020) 126:2120–31. doi: 10.1002/cncr.32756 32125712

[7] Herman JM Chang DT Goodman KA Dholakia AS Raman SP Hacker-Prietz A. Phase 2 multi-institutional trial evaluating gemcitabine and stereotactic body radiotherapy for patients with locally advanced unresectable pancreatic adenocarcinoma. Cancer (2015) 121:1128–37. doi: 10.1002/cncr.29161 PMC4368473 25538019

[8] Mahadevan A Miksad R Goldstein M Sullivan R Bullock A Buchbinder E. Induction gemcitabine and stereotactic body radiotherapy for locally advanced nonmetastatic pancreas cancer. Int J Radiat Oncol Biol Phys (2011) 81:615–22. doi: 10.1016/j.ijrobp.2011.04.045 21658854

[9] Mahadevan A Moningi S Grimm J Li XA Forster KM Palta M. Maximizing tumor control and limiting complications with stereotactic body radiation therapy for pancreatic cancer. Int J Radiat Oncol Biol Phys (2021) 110:206–16. doi: 10.1016/j.ijrobp.2020.11.017 33358561

[10] Oar A Lee M Le H Hruby G Dalfsen R Pryor D. Australasian Gastrointestinal trials group (AGITG) and trans-Tasman radiation oncology group (TROG) guidelines for pancreatic stereotactic body radiation therapy (SBRT). Pract Radiat Oncol (2020) 10:e136–46. doi: 10.1016/j.prro.2019.07.018 31761541

[11] Lacobuzio-Donahue CA Fu B Yachida S Luo M Abe H Henderson CM. DPC4 gene status of the primary carcinoma correlates with patterns of failure in patients with pancreatic cancer. J Clin Oncol (2009) 27:1806–13. doi: 10.1200/JCO.2008.17.7188 PMC2668706 19273710

[12] Pishvaian MJ Blais EM Brody JR Lyons E DeArbeloa P Hendifar A. Overall survival in patients with pancreatic cancer receiving matched therapies following molecular profiling: a retrospective analysis of the know your tumor registry trial. Lancet Oncol (2020) 21:508–18. doi: 10.1016/S1470-2045(20)30074-7 PMC7453743 32135080

[13] Hammel P Huguet F Van Laethem JL Goldstein D Glimelius B Artru P. Effect of chemoradiotherapy vs chemotherapy on survival in patients with locally advanced pancreatic cancer controlled after 4 months of gemcitabine with or without erlotinib the LAP07 randomized clinical trial. JAMA - J Am Med Assoc (2016) 315:1844–53. doi: 10.1001/jama.2016.4324 27139057

[14] Petrelli F Comito T Ghidini A Torri V Scorsetti M Barni S. Stereotactic body radiation therapy for locally advanced pancreatic cancer: A systematic review and pooled analysis of 19 trials. Int J Radiat Oncol Biol Phys (2017) 97:313–22. doi: 10.1016/j.ijrobp.2016.10.030 28068239

[15] Toesca DAS Ahmed F Kashyap M Baclay JRM von Eyben R Pollom EL. Intensified systemic therapy and stereotactic ablative radiotherapy dose for patients with unresectable pancreatic adenocarcinoma. Radiother Oncol (2020) 152:63–9. doi: 10.1016/j.radonc.2020.07.053 32763253

[16] Arcelli A Guido A Buwenge M Simoni N Mazzarotto R Macchia G. Higher biologically effective dose predicts survival in SBRT of pancreatic cancer: A multicentric analysis (PAULA-1). Anticancer Res (2020) 40:465–72. doi: 10.21873/anticanres.13975 31892602

[17] Brunner TB Nestle U Adebahr S Gkika E Wiehle R Baltas D. Simultan integrierte protektion: Ein neues konzept für die hochpräzisionsbestrahlung. Strahlentherapie und Onkol (2016) 192:886–94. doi: 10.1007/s00066-016-1057-x PMC5122615 27757502

[18] Rossi G Simoni N Paiella S Rossi R Venezia M Micera R. Risk adapted ablative radiotherapy after intensive chemotherapy for locally advanced pancreatic cancer. Front Oncol (2021) 11:662205. doi: 10.3389/fonc.2021.662205 33959509 PMC8093383

[19] Mellon EA Hoffe SE Springett GM Frakes JM Strom TJ Hodul PJ. Long-term outcomes of induction chemotherapy and neoadjuvant stereotactic body radiotherapy for borderline resectable and locally advanced pancreatic adenocarcinoma. Acta Oncol (Madr) (2015) 54:979–85. doi: 10.3109/0284186X.2015.1004367 25734581

[20] Simoni N Micera R Paiella S Guariglia S Zivelonghi E Malleo G. Hypofractionated stereotactic body radiation therapy with simultaneous integrated boost and simultaneous integrated protection in pancreatic ductal adenocarcinoma. Clin Oncol (2021) 33:e31–8. doi: 10.1016/j.clon.2020.06.019 32682686

[21] Mazzarotto R Simoni N Guariglia S Rossi G Micera R De Robertis R. Dosimetric feasibility study of dose escalated stereotactic body radiation therapy (SBRT) in locally advanced pancreatic cancer (LAPC) patients: It is time to raise the bar. Front Oncol (2020) 10:600940. doi: 10.3389/fonc.2020.600940 33392093 PMC7773844

[22] Hall WA Small C Paulson E Koay EJ Crane C Intven M. Magnetic resonance guided radiation therapy for pancreatic adenocarcinoma, advantages, challenges, current approaches, and future directions. Front Oncol (2021) 11:628155. doi: 10.3389/fonc.2021.628155 34046339 PMC8144850

[23] Hassanzadeh C Rudra S Bommireddy A Hawkins WG Wang-Gillam A Fields RC. Ablative five-fraction stereotactic body radiation therapy for inoperable pancreatic cancer using online MR-guided adaptation. Adv Radiat Oncol (2021) 6:1–8. doi: 10.1016/j.adro.2020.06.010 PMC7897757 33665480

[24] Rudra S Jiang N Rosenberg SA Olsen JR Roach MC Wan L. Using adaptive magnetic resonance image-guided radiation therapy for treatment of inoperable pancreatic cancer. Cancer Med (2019) 8:2123–32. doi: 10.1002/cam4.2100 PMC6536981 30932367

[25] Chuong MD Bryant J Mittauer KE Hall M Kotecha R Alvarez D. Ablative 5-fraction stereotactic magnetic resonance–guided radiation therapy with on-table adaptive replanning and elective nodal irradiation for inoperable pancreas cancer. Pract Radiat Oncol (2021) 11:134–47. doi: 10.1016/j.prro.2020.09.005 32947042

[26] Lischalk JW Burke A Chew J Elledge C Gurka M Marshall J. Five-fraction stereotactic body radiation therapy (SBRT) and chemotherapy for the local management of metastatic pancreatic cancer. J Gastrointest Cancer (2018) 49:116–23. doi: 10.1007/s12029-016-9909-2 28044263

[27] Rosati LM Herman JM. Role of stereotactic body radiotherapy in the treatment of elderly and poor performance status patients with pancreatic cancer. J Oncol Pract (2017) 13:157–66. doi: 10.1200/JOP.2016.020628 28282277

