# Supplementary figures and images for: Consolidatory ablative stereotactic body radiation therapy after induction chemotherapy for unresectable pancreatic cancer: A single center experience
Source: Front Oncol. 2022 Nov 18;12:974454. doi: 10.3389/fonc.2022.974454 (PMC9733675; doi:10.3389/fonc.2022.974454)

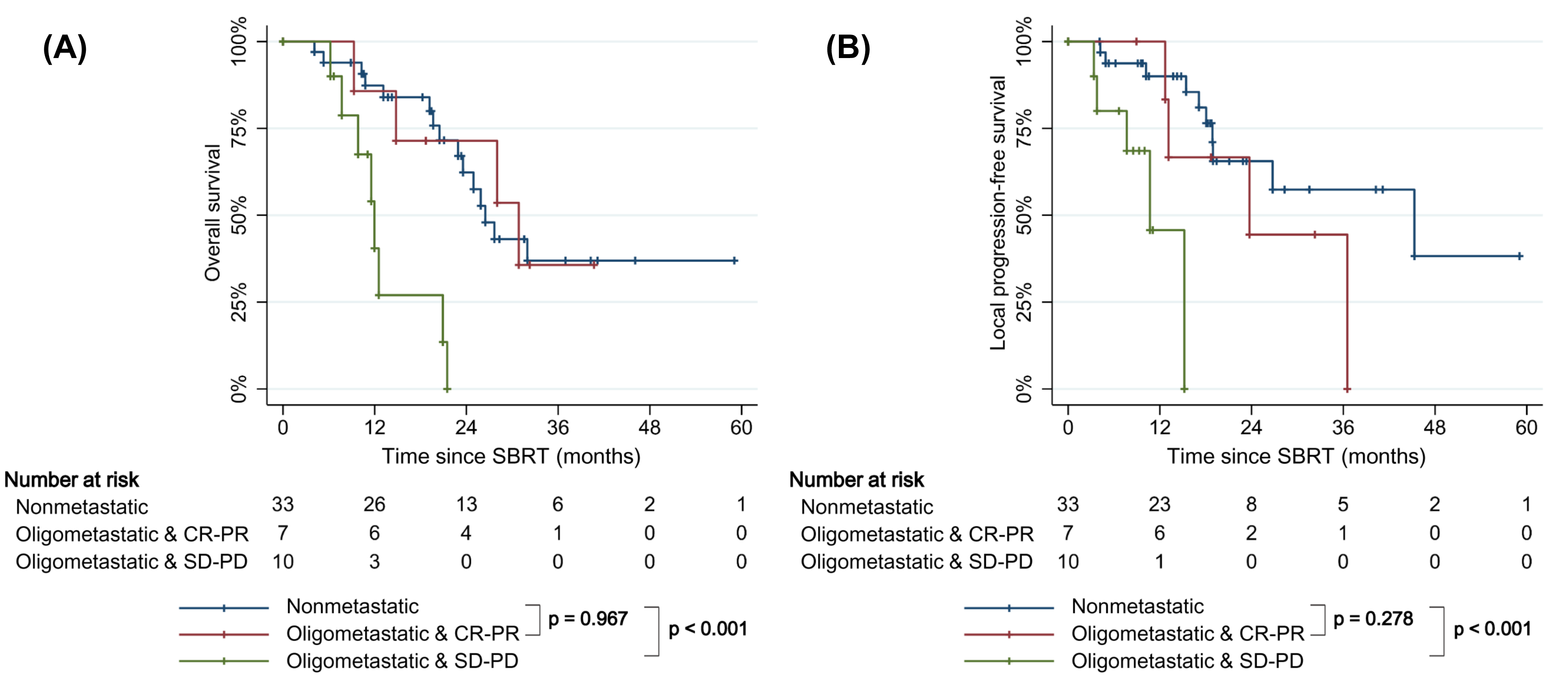

Supplement: Supplementary Figure 1 — Kaplan-Meier curves of (A) overall survival, and (B) local progression-free survival according to metastatic status and overall response to the induction chemotherapy. SBRT, stereotactic body radiotherapy. [file Image_1.tiff]

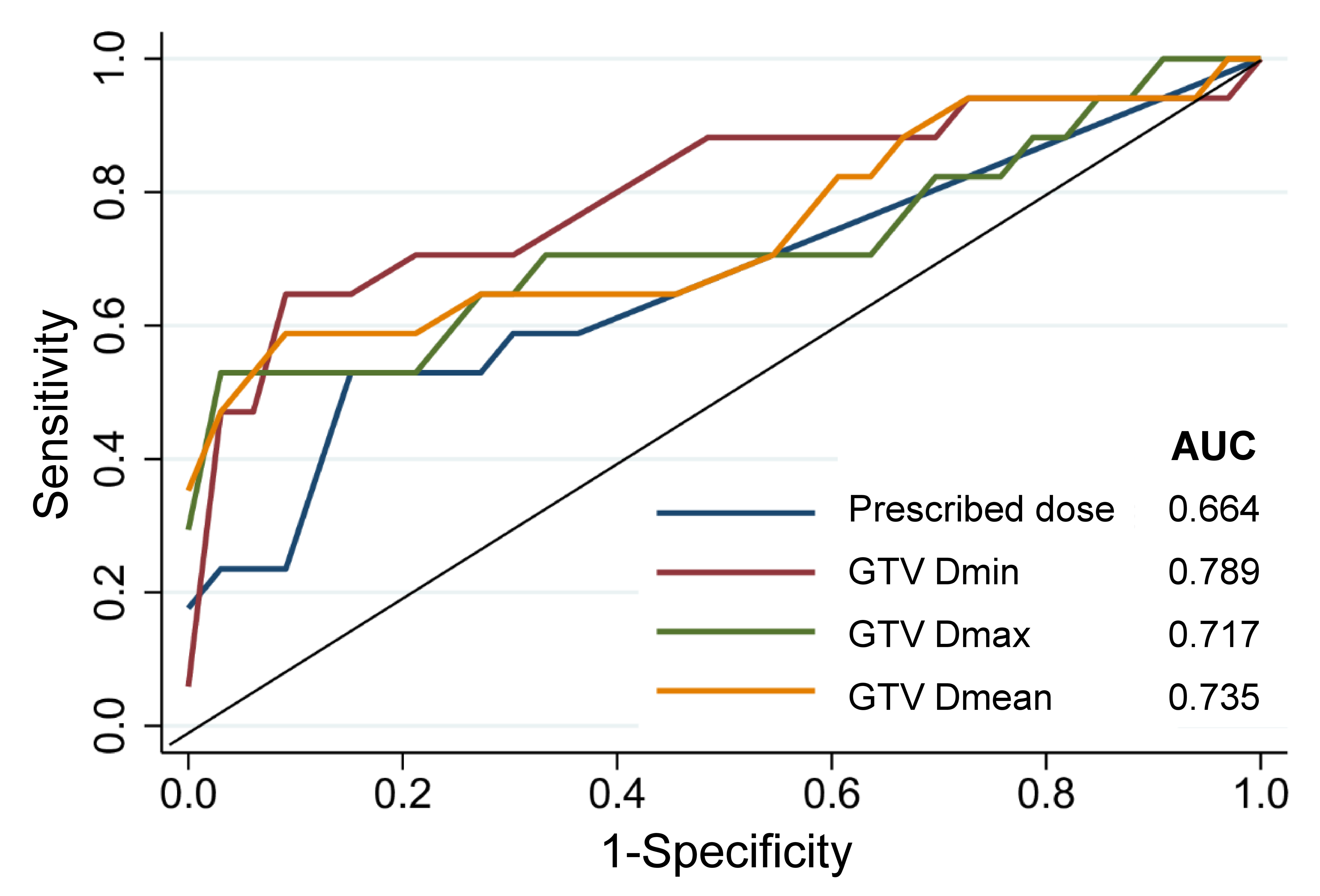

Supplement: Supplementary Figure 2 — Comparison of receiver operating characteristic (ROC) curves of dosimetric parameters for predicting the 3-year incidence of local progression. GTV, gross tumor volume; AUC, area under curve.GTV Dmin, the minimum dose absorbed by 1cc of the GTV; GTV Dmax, the maximum dose absorbed by 1cc of the GTV; GTV Dmean, mean dose absorbed by the GTV. [file Image_2.tiff]
